# Genome-wide identification of selection signals in fat-tailed and thin-tailed sheep populations

**DOI:** 10.3389/fgene.2025.1581914

**Published:** 2025-10-17

**Authors:** Lei Gao, Yiyuan Zhang, Bin Zhang, Weifeng Peng, Yucheng Liu, Zhenliang Zhang, Jingjing Wang, Pengcheng Wan, Hua Yang, Zongsheng Zhao

**Affiliations:** ^1^ College of Animal Science and Technology, Shihezi University, Shihezi, Xinjiang, China; ^2^ State Key Laboratory of Sheep Genetic Improvement and Healthy Production, Xinjiang Academy of Agricultural and Reclamation Science, Shihezi, China; ^3^ College of Life Science and Agronomy, Zhoukou Normal University, Zhoukou, China

**Keywords:** sheep, selection signatures, fat-tail, whole-genome resequencing, gene ontology

## Abstract

**Introduction:**

In the evolutionary context of sheep, the development of fat tails represents an adaptive survival mechanism in response to varying food availability. Despite food resource instability, sheep store energy by accumulating tail fat to survive periods of famine. This energy storage function remains present in domesticated sheep, serving as a key evolutionary reason for the formation of sheep tail fat.

**Methods:**

Here, we conducted whole-genome resequencing of 555 sheep samples (30 samples were newly sequenced and 525 were retrieved from published data) globally to investigate selection signatures associated with fat-tailed traits using Fixation Index (F_ST_), Nucleotide diversity (π), cross-population composite likelihood ratio (XP-CLR), and runs of homozygosity (ROH) methods.

**Result and discussion:**

Our examination of selection signatures in Fat-tailed and Thin-tailed Sheep Populations identified 32 candidate genes, with 6 genes (*PDGFD*, *BMP2*, *GLIS1*, *LIPE*, *MSRB3*, and *TBX15*) implicated in fat accumulation and lipid metabolism. Notably, 8 significant Gene Ontology terms (mesenchymal cell differentiation, positive regulation of ERK1 and ERK2 cascades, hormone metabolic process, nucleocytoplasmic transport, regulation of hormone levels, response to growth factor, regulation of canonical Wnt signaling pathway, and tissue morphogenesis) may play a role in fat deposition and tail fat development. These results will provide molecular targets for low-fat sheep breeding and enhance economic returns in sheep farming.

**Conclusion:**

This study will play a crucial role in environmental adaptation and product development, comprehensively driving the development of the sheep farming industry and enhancing economic benefits.

## Introduction

Sheep are among the earliest domesticated livestock, playing crucial roles in the production of meat, wool, and milk (Tian et al., 2024; Peng et al., 2022). Sheep are one of the major livestock species in China, particularly important in arid and grassland regions. Their presence not only supports the livelihoods of countless herding families in provinces such as Inner Mongolia, Xinjiang, and Qinghai, but also plays a pivotal role in the regional ecological balance. China, with a long history of domestication and breeding, boasts a rich diversity of sheep breeds that are affected by human migration and climate (Abied et al., 2020). Additionally, sheep breeds can be classified by the type of tail fat deposition and tail morphology, including short thin-tailed, long thin-tailed, short fat-tailed, long fat-tailed, and fat-rumped sheep (Li et al., 2020). Representative Chinese breeds include short fat-tailed sheep like Small-tailed Han sheep and Mongolian sheep (Liu et al., 2016), long fat-tailed sheep like Tan sheep, Large-tailed Han sheep, and Tong sheep, short thin-tailed sheep like Tibetan sheep and Hanzhong fine wool sheep, and predominantly long thin-tailed breeds, including most fine wool and semi-fine wool sheep (Liu et al., 2021). Fat-rumped sheep have large tails with fat deposits in the rump, as seen in breeds such as Altay sheep and Kazakh sheep (Zhu et al., 2020).

It is widely believed that fat-tailed sheep have evolved from thin-tailed sheep through long-term natural selection in extremely harsh geographical and climatic conditions (Caiye et al., 2023). The fat-tail trait enables sheep to store sufficient energy, which is crucial for survival in times of food scarcity. In most regions worldwide, fat-tailed sheep are generally more adaptable to harsh environments compared to other tail types in the same area (Kalds et al., 2022). Phenotypic analyses of growth, development, and production of sheep in China, along with factors related to geographical and climatic environments, reveal that climate types significantly influence the distribution of thin-tailed and fat-tailed sheep (Abied et al., 2020). Short fat-tailed and fat-rumped sheep are primarily found in northern regions, where the fat-tail trait, allowing for greater energy storage, has evolved through natural selection in relatively harsh environments.

In contrast, southern regions are mainly populated by short thin-tailed sheep (Qi et al., 2024). The high proportion of non-terminal branched-chain fatty acids in sheep tail fat, along with its unique lipid metabolism process, suggests that genes or regulatory pathways controlling tail fat deposition may differ from those involved in fat deposition in other body parts (Alves et al., 2013). The specific genetic mechanisms underlying tail fat deposition remain unclear, with only a partial identification of candidate genes and nucleotide fragments associated with tail phenotype. Genome-wide association studies in Iranian fat-tailed and thin-tailed sheep have identified highly homozygous single-nucleotide polymorphism (SNP) loci on chromosomes X and 5 in fat-tailed sheep, and on chromosome 7 in thin-tailed sheep, suggesting a potential association of these SNPs with sheep fat deposition and meat quality (Moradi et al., 2012). Further screening has revealed a homozygous SNP site on the intron of the androgen receptor (*AR*) gene on the X chromosome, showing high fat-tail deposition efficiency in Altay sheep and Hu sheep, while displaying significant polymorphism in the thin-tailed Chinese Merino and Suffolk sheep, indicating a potential correlation of this SNP site with tail fat traits (Zhang et al., 2021).

In addition, by comparing the differential expression of whole-genome mRNA and conducting corresponding KEGG analysis in the fat tissues of fat-tailed Tibetan sheep and thin-tailed Dorset sheep, it was uncovered that genes with significantly altered expression levels were mostly related to lipid metabolism (Zhang et al., 2021). Using restriction fragment length polymorphism (RFLP) analysis, researchers detected two SNP loci on the X chromosome in Altay sheep and Hu sheep, which exhibit significant differences in tail fat deposition efficiency. The distribution of SNPs at the 59,571,364 and 59,912,586 loci between sheep with significantly different tail types suggests that these two SNPs could serve as genetic markers for breeding sheep with high or low tail fat content (Zhang et al., 2021). Through whole-genome selection signal detection based on the population differentiation index F_ST_ of SNPs in Mongolian sheep and Tibetan sheep, researchers found that the expression levels of the fat deposition-related genes *PPARG* and *PDGFD* in the fat-tailed Hulun Buir sheep (divided into long-tailed and short-tailed breeds) were significantly higher than those in the short-thin-tailed Tibetan sheep (Dong et al., 2020). Additionally, within the long-tailed and short-tailed breeds, there were expression level differences in the *PPARG* gene that were positively correlated with tail fat deposition traits (Cui et al., 2022). Accumulating evidence indicates that multiple factors, including genetic variations, hormonal regulation, and environmental conditions, interact to modulate fat tail deposition in sheep. Previous studies mainly focused on individual genes or pathways, lacking a comprehensive understanding of the genetic-environmental interactions. Our study aims to bridge these gaps by integrating multiple datasets to uncover novel regulatory mechanisms underlying fat tail deposition in sheep.

The Kazakh sheep are initially from the northern foothills of the Tianshan Mountains and the southern foothills of the Altai Mountains. The region experiences dramatic seasonal climate variations, characterized by hot summers, cold winters, unpredictable spring temperatures, and rapid temperature drops in autumn. The development of Kazakh sheep is closely tied to the selective breeding practices of local ethnic groups and the stable ecological conditions (Zhu et al., 2023). Researchers at Xinjiang Academy of Agricultural Sciences conducted a breeding program by crossing multi-fetal Suffolk sheep (♂) as the paternal line with Kazakh sheep (♀) as the maternal line (Yang L. et al., 2024). Through decades of selection, they developed the hybrid F1 generation, which underwent further breeding to produce the F2 and F3 generations. The hybrid offspring exhibited phenotypic differences, notably reduced tail fat deposition (less than one-third that of Kazakh sheep). The populations of the hybrid F1, F2, and F3 generations currently consist of approximately 200 individuals each. The study involved sequencing 10 Kazakh sheep, 10 prolific Suffolk sheep, and 10 hybrid F2 generation individuals, along with resequencing data from 525 sheep previously published. This comprehensive analysis aimed to investigate the population structure and selection signals of different sheep tail types. The results are expected to elucidate the genetic regulatory mechanisms underlying tail fat deposition in sheep and offer valuable genetic resources for the molecular breeding of tail fat traits in fat-tailed sheep breeds.

## Methods

### Data

Thirty sheep individuals, consisting of 10 Kazakh sheep, 10 prolific Suffolk sheep, and 10 hybrid F2 generation individuals, were sourced from the sheep farm affiliated with the Xinjiang Academy of Agricultural and Reclamation Science. The collection site of the female Kazakh sheep selected for the samples is the 181th Herd Sheep Farm, which is the birthplace of the local Kazakh sheep breed. The male prolific Suffolk sheep selected for the samples is a new variety that the Xinjiang Academy of Agricultural Sciences independently bred. The population of Kazakh sheep and prolific Suffolk sheep has reached 5,000, with about 400 in the F2 generation of hybridization. We collected 5 mL blood samples from the jugular veins of each sheep, treating them with EDTA-Na2 before promptly storing them at −20 °C for future processing. DNA extraction from the blood samples was performed using the TIANamp Blood DNA Kit (TIANGEN, China), following the manufacturer’s detailed instructions precisely. Following extraction, we assessed the quality of genomic DNA using 1% agarose gel electrophoresis and precisely measured its concentration and purity with the reliable NanoDrop 2000 spectrophotometer.

### Read alignment and variable annotation

Moreover, to expand the sample size and thus boost the analytical reliability for both fat-tailed and thin-tailed groups, we incorporated 525 previously published sheep resequencing datasets (Li et al., 2020), with detailed information provided in Supplementary Table S1. The valid sequencing data were mapped to the sheep reference genome (Oar_v4.0) using BWA (v.0.7.12) with the parameters “mem–t 4 –k 32 -M” (Li and Durbin, 2009). We enthusiastically utilized SAMtools (version 1.2) to detect Single Nucleotide Polymorphisms (SNPs), applying parameters like “mpileup–m 2 –F 0.002 -d 1,000” (Li et al., 2009). To ensure accuracy in variant calling, we thoughtfully incorporated targeted filtering criteria to minimize errors. Variant sites with a Quality by Depth (QD) value less than 2.0, a Mapping Quality (MQ) value less than 20, and a Fisher Strand (FS) value greater than 60.0 were removed. After this filtering process, the remaining variants were annotated using ANNOVAR version 21 (Yang H. et al., 2024). We applied quality filtering to the SNP dataset using VCFtools v0.1.17 (Danecek et al., 2011) excluding SNPs that satisfied any of the following criteria: (1) a call rate of 90% or lower; (2) a minor allele frequency (MAF) of 0.05 or less; or (3) a mean maximum depth below 3 or above 30. Following this quality control process, a total of 11,852,938 SNPs were kept for subsequent analyses.

### Population genetic structure and LD decay analysis

We enthusiastically used the neighbor-joining (NJ) method from the Phylogeny Inference Package (PHYLIP) to create a phylogenetic tree, utilizing high-quality, filtered single-nucleotide polymorphisms (SNPs) as outlined by Felsenstein (1989). We enthusiastically explored the population structure through cluster analysis using ADMIXTURE (version 1.3.0, Alexander and Lange, 2011). It was an engaging process, as we carefully set our parameters to range from 2 to 6, executing the command “for K in 2–6, execute admixture--cv sheep.” bed | tee log.out, done” was implemented, with a cap of 10,000 iterations. In addition, to gain further insights into the genetic relationships among the samples, Principal Component Analysis (PCA) was conducted on the 555 samples. We employed the EIGENSOFT package, specifically version 7.2.1, to perform this analysis (Price et al., 2006). We used the PopLDdecay software (version 3.4.3) (Zhang et al., 2019) to evaluate the linkage disequilibrium (LD) decay in sheep breeds. The analysis was conducted using the software’s default parameters.

### Selection signals analysis

The 555 sheep were categorized into two distinct groups: the fat-tailed group (including sheep with fat rumps, fat tails, long fat tails, and short fat tails) and the thin-tailed group (including sheep with long thin tails, short thin tails, and thin tails) (Supplementary Table S1). To screen for potential genomic regions, we conducted a genome-wide analysis of F_ST_ and π ratio distributions using a window-based strategy (50 kb windows with 25 kb intervals) (Danecek et al., 2011; Peng et al., 2024a). For statistical normalization, Z-score transformation was applied to F_ST_ values, while π ratios underwent log2 conversion. Candidate selection signals were defined as overlapping windows featuring the extreme values for both metrics (He et al., 2024; Yang et al., 2025). These outlier regions were then mapped to corresponding SNPs and annotated with relevant genes. In addition to the initial screening, we conducted sweep detection using the XP-CLR algorithm (version 1.0) (Chen et al., 2010). The analysis incorporated SNPs with <10% missing data (“-max-missing 0.9”) and utilized specific parameters: 1-kb grid spacing, 200-SNP maximum window size, and reduced weight for highly correlated SNPs (r^2^ > 0.95) through (-w1 0.005 200 2000 2 -p0 0.95). Regions displaying top 1% genome-wide XP-CLR scores were designated as strong selective sweep candidates (Lv et al., 2021; Peng et al., 2025). To further identify genomic regions most frequently associated with ROH, we calculated the percentage of SNP occurrences in ROH by counting the number of times each SNP appeared in ROH across individuals using PLINK (Purcell et al., 2007) and a length threshold of ROH >0.5 Mb. This percentage was then plotted against the SNP’s chromosomal position. A SNP was considered indicative of a potential ROH hotspot if its occurrence percentage exceeded 20%. Adjacent SNPs with ROH occurrence proportions above this 20% threshold formed continuous genomic segments termed ROH islands (Peng et al., 2024b).

### GO enrichment analysis

In addition, overlap genes identified by three methods (F_ST_, ROH, and XP-CLR) were subjected to Gene Ontology (GO) enrichment analysis using DAVID 6.8 (Huang et al., 2009; Lukic et al., 2023). The significantly enriched GO terms (*P* < 0.05) were visualized using R software (version 4.2.1).

## Results

### Sequencing and mapping

Thirty Suffolk sheep were tested on the Illumina HiSeq 2500 platform, resulting in a total of 5,245.855 gigabytes (Gb) of raw data. After a stringent filtering process, 5,236.338 Gb of clean reads were obtained. The quality of these clean reads was high, as evidenced by a Q20 value of at least 95.73% and a Q30 value of at least 89.69%. The GC content of the clean reads ranged from 43.09% to 46.62%. The clean reads were then mapped to the sheep reference genome (Oar_v4.0). The genome mapping rate varied from 98.65% to 99.32%, suggesting a strong alignment to the reference. When considering the average coverage depth across three sheep breeds, it was approximately 13.21×. Moreover, the proportion of the genome with at least 1 × average coverage exceeded 98.06%, and the proportion with at least 4 × average coverage exceeded 94.29%. These high-coverage values are indicative of the accuracy and reliability of the sequencing data. To further analyze the data, summary information was extracted from the input binary alignment/map (BAM) files using SAMTools. This tool was used to compute genotype likelihoods and convert the data into the binary variant call format (BCF). Subsequently, the ANNOVAR software was employed. It carried out the functional annotation of gene mutations and transformed the data into the Variant Call Format (VCF), which is suitable for subsequent in-depth genetic analyses. After mapping and SNP calling, a total of 11, 852,938 SNPs were identified from the 555 sheep samples and can be effectively utilized to uncover genetic variations and their potential biological implications in the studied sheep breeds.

### Population genetic structure and LD decay

**FIGURE 1:**
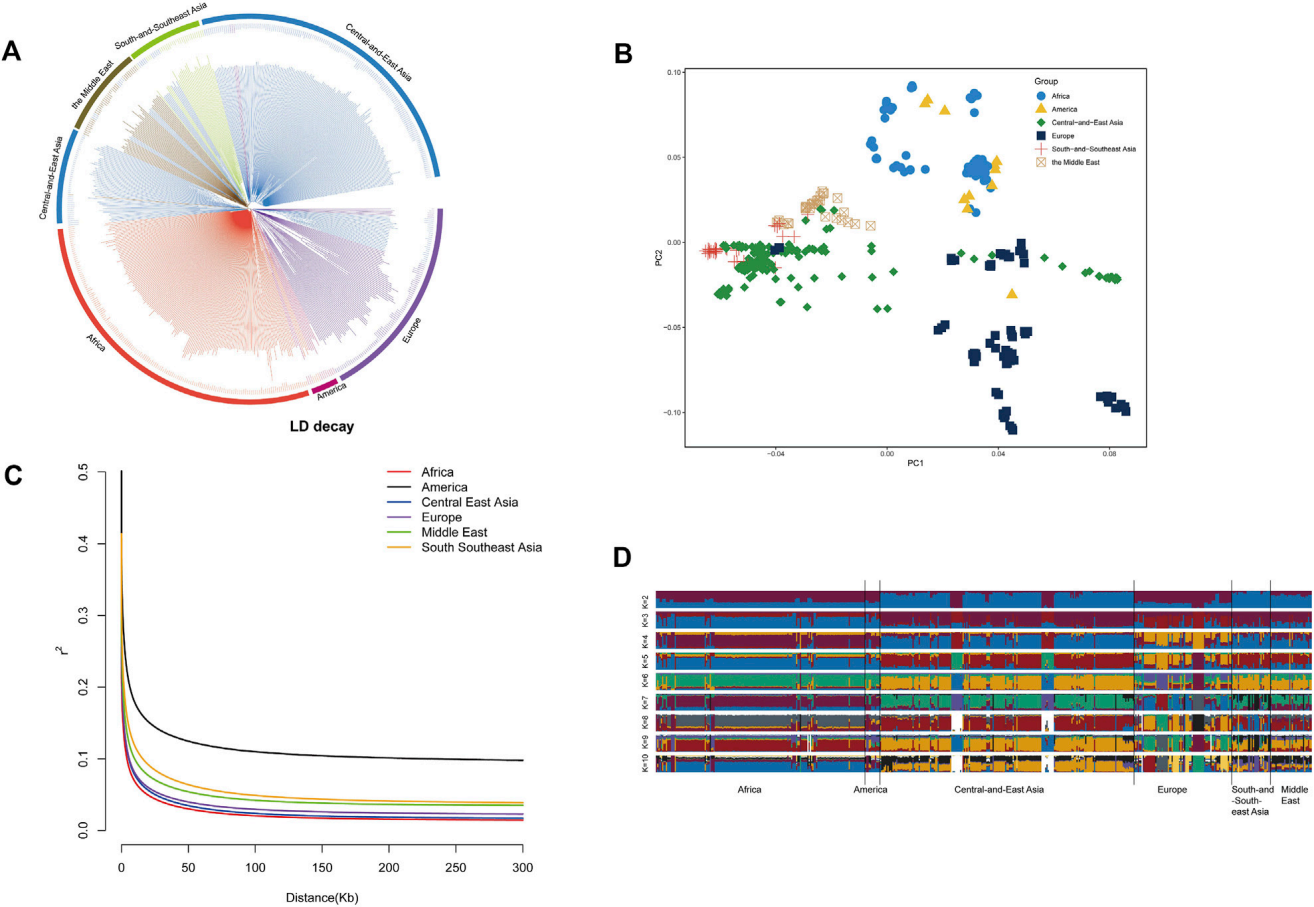
Population structure analysis: **(A)** a Neighbor-Joining (NJ) tree of 555 sheep individuals based on p-distances, **(B)** Principal Component Analysis of the 555 sheep individuals, **(C)** population structure analysis using ADMIXTURE with K = 2-10, and **(D)** the decay of *r* ^*2*^ with pair-wise SNP marker distances in sheep populations from Africa, America, Central-and-East Asia, Europe, South and Southeast Asia, and Middle East.

The population genetic structure of 555 sheep was classified into 6 distinct geographic regions: the Middle East, Central and East Asia, South and Southeast Asia, Europe, America, and Africa (Figures 1A–C). The results showed that there was no apparent genetic differentiation between Asian geographical regions (including the Middle East, Central and East Asia, and South and Southeast Asia), suggesting that the sheep in these regions are influenced by human activities and there was an exchange of genetic material. In addition, sheep in the Central and East Asia region exhibited specific European lineages. For example, Chinese Merino Sheep clustered with European sheep populations. In contrast, African White Dorper sheep clustered with those in the Americas (Figure 1A). In addition, the American sheep population exhibited a high level of linkage disequilibrium (LD). This high LD level can be attributed to the intensive artificial selection and breeding strategies implemented in the American sheep industry over the past century. For instance, to meet the demand for fast growth and high meat production, breeders have consistently conducted directional selection and inbreeding mating, which has led to a reduction in genetic recombination opportunities in specific genomic regions, thereby increasing the degree of LD. The limitation of this study lies in the small sample size, which fails to include the original American breeds. Future research should expand the sample scope and combine genome-wide selection signal analysis to more accurately analyze the evolutionary mechanism of LD. By contrast, the African sheep population showed the lowest degree of LD (Figure 1D), indicating that it has been less influenced by artificial selection and commercial breeding practices.

### Selection signals between fat-tailed and thin-tailed sheep populations

**FIGURE 2:**
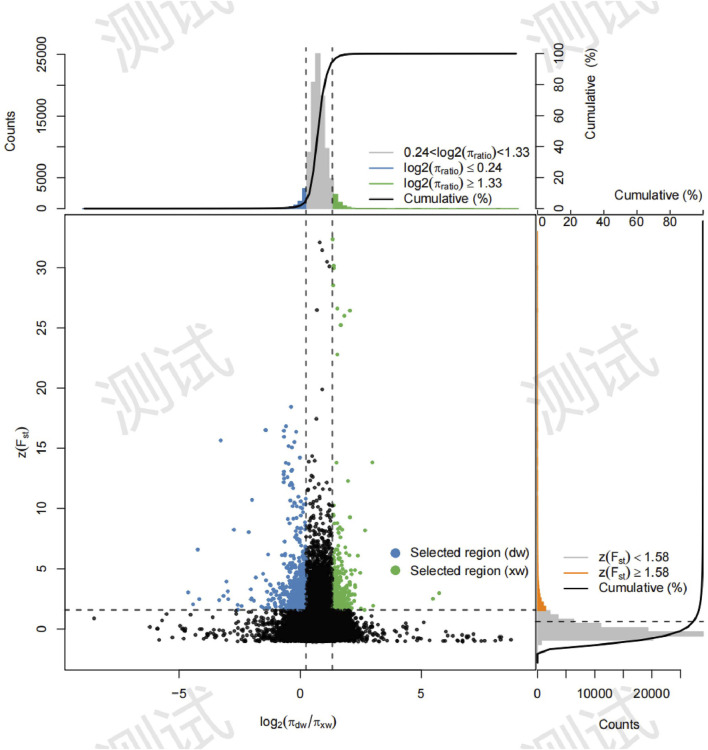
Selection signatures between fat-tailed and thin-tailed sheep populations using Fst-Pi method.

**FIGURE 3:**
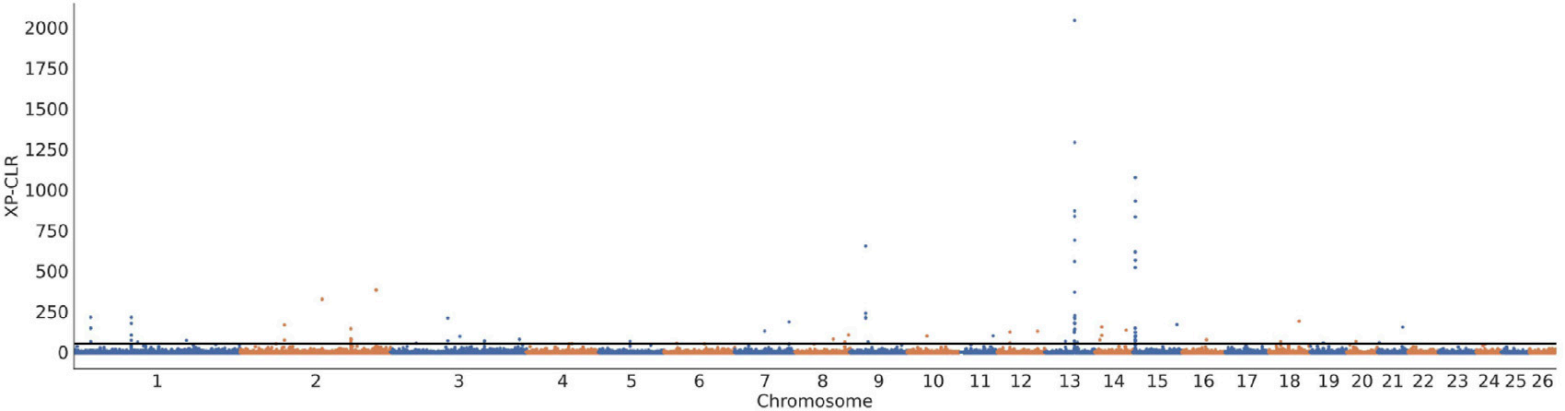
Selection signatures between fat-tailed and thin-tailed sheep populations using XP-CLR test.

**FIGURE 4:**
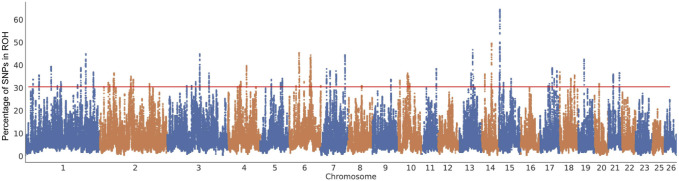
Manhattan plots of the distribution of ROH in fat-tailed breeds. The x-axis is the autosome number and the y-axis shows the frequency (%) at which each SNP was observed in ROH across individuals.

**FIGURE 5:**
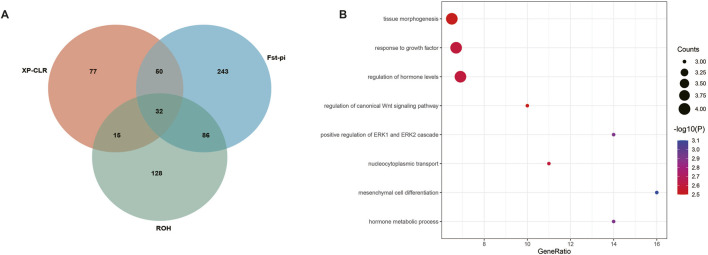
Genes and GO terms identified by Fst-Pi, XP-CLR, and ROH test. **(A)** Venn diagram for genes identified by Fst-Pi, XP-CLR, and ROH test. **(B)** Top 8 GO terms identified based on 32 candidate genes.

Three approaches (F_ST_-Pi, XP-CLR, and ROH) were used to identify selection signatures between Fat-tailed and Thin-tailed sheep populations. According to F_ST_ values with z (F_ST_) > 1.58 and π ratio thresholds with log2(πdw/πxw) < 0.24, 411 candidate genes were identified (Figure 2; Supplementary Table S2). For the XP-CLR analysis, 169 genes were pinpointed based on the top 1% of XP-CLR values (Figure 3; Supplementary Table S3). In the ROH tests, using a threshold exceeding 30%, 256 genes were identified, with chromosome 15 harboring the highest proportion of ROH segments in animals (Figure 4; Supplementary Table S4). 32 overlapping genes were identified by the three methods (Figure 5A; Supplementary Table S5). Among these 32 genes, several have been explicitly reported in molecular mechanism studies of ovine caudal fat deposition and are implicated in adipocyte differentiation, regulation of lipid metabolism, or selection signatures for tail fat morphology. For instance, *PDGFD* inhibits adipose tissue expansion by activating the *PDGFRβ* pathway, exhibits higher expression levels in thin-tailed sheep breeds, and thereby negatively regulates caudal fat deposition. *BMP2* regulates adipocyte differentiation and lipid droplet formation, with its expression level showing a significant negative correlation with caudal fat mass. Additionally, *GLIS1* acts as a pro-adipogenic factor, influencing mesoderm cell differentiation and caudal fat deposition. Furthermore, novel candidate genes were identified, including *TBX15*, which is involved in embryonic development regulation and known to play a role in brown adipose tissue, yet its function in ovine caudal fat remains unvalidated; and *LIPE*​ (Hormone-sensitive lipase), which plays a well-established role in lipolysis, but its expression and function in ovine caudal adipose tissue have not been specifically studied or reported.

### GO enrichments and KEGG analysis

Functional annotation of the 32 overlapping genes revealed eight significant Gene Ontology (GO) terms (Figure 5B; Table 1). Key terms associated with established adipogenic mechanisms include Mesenchymal cell differentiation (involving *BMP2*, *NOTCH1*, and *ALDH1A2*), which mediates precursor adipocyte maturation (Zhao et al., 2024); Response to growth factor (featuring *BMP2*, *MEGF8*, *NOTCH1*, and *PDGFD*), known to promote adipocyte differentiation through signaling cascades (Xu et al., 2023); and Tissue morphogenesis (including *BMP2*, *MEGF8*, *NOTCH1*, and *ALDH1A2*) (Jin et al., 2025), implicated in caudal fat development via vascular remodeling and adipose tissue spatial organization. Notably, novel GO terms with potential but unexplored roles in ovine fat metabolism were also identified. Positive regulation of ERK1 and ERK2 cascades modulates cell differentiation in contexts such as pulmonary fibrosis and chondrogenesis via transcription factor phosphorylation (e.g., *Runx2*); however, its functional impact on ovine adipogenesis remains uncharacterized. Similarly, the Regulation of canonical Wnt signaling—demonstrated in humans and mice to suppress preadipocyte differentiation by inhibiting *PPARγ* and *C/EBPα*—exhibits analogous regulatory potential for negative lipid deposition feedback in ovine caudal fat (Wang et al., 2025), yet, direct functional evidence remains lacking. Collectively, these findings highlight conserved adipogenic pathways while underscoring significant species-specific knowledge gaps, particularly concerning ERK/Wnt-mediated regulatory mechanisms governing ovine tail fat deposition.

**TABLE 1:** 8 GO terms identified based on 32 candidate genes.

Description	-log10(P)	GeneRatio	Counts	Genes
Mesenchymal cell differentiation	3.1	16	3	BMP2|NOTCH1|ALDH1A2
Positive regulation of ERK1 and ERK2 cascade	2.9	14	3	BMP2|NOTCH1|PDGFD
Hormone metabolic process	2.9	14	3	LIPE|ALDH1A2|PAPSS2
Nucleocytoplasmic transport	2.6	11	3	NOTCH1|NDC1|NUP85
Regulation of hormone levels	2.6	6.9	4	BMP2|LIPE|ALDH1A2|PAPSS2
Response to growth factor	2.6	6.7	4	BMP2|MEGF8|NOTCH1|PDGFD
Regulation of canonical Wnt signaling pathway	2.5	10	3	BMP2|GNAQ|NOTCH1
Tissue morphogenesis	2.5	6.5	4	BMP2|MEGF8|NOTCH1|ALDH1A2

## Discussion

The population structure and genetic diversity of sheep play a crucial role in assessing their genetic resources, which in turn have a bearing on the utilization and conservation of these resources. After Principal Component Analysis (PCA), Neighbor-Joining tree construction, and ADMIXTURE analysis, clear differentiations were observed among Asian (Central and East Asia, South and Southeast Asia, and the Middle East), European, and African sheep, and sheep from the American region are relatively close to Europe, and it has no obvious geographical features. Additionally, it has been noted that there is genetic interaction among sheep populations on different continents, primarily due to the impact of human activities following the domestication of sheep, particularly the use of certain commercial sheep breeds (Figures 1A–C). Especially in the Asian region, including Central-East Asia, South-Southeast Asia, and the Middle East, there have been more genetic exchanges. Because these regions are adjacent to each other, historical human activities have been frequent. For instance, during the early Neolithic Age, sheep spread from the Near East to East Asia via the Eurasian communication route, with their migration routes overlapping with historical trade routes, such as the Silk Road (Yang H. et al., 2024). The Middle East (such as the Fertile Crescent) is the origin of sheep domestication, whose genetic components gradually spread to Central Asia, South Asia, and Southeast Asia through human migration (Daly et al., 2025). Besides, we found that Chinese Merino Sheep from China were found clustered together with European sheep breeds (Figure 1A). This is because the breeding of Chinese Merino sheep is usually achieved by crossbreeding European Merino sheep (such as Spanish Merino or German Merino) with native Chinese sheep (such as Gansu mountain fine-wool sheep) (Li et al., 2023). This directed hybridization strategy directly introduced genomic fragments of European Merino sheep, resulting in a genetic structure closer to that of European breeds. LD decay revealed that African populations displayed rapid LD decay and minimal LD levels, reflecting heightened genetic recombination and reduced selection pressure. Conversely, the American population exhibited the slowest LD decay and the highest LD levels, indicating strong artificial selection and low genetic diversity (Figure 1D).

The identification of lipid selection signals in the sheep tail genome represents a significant advancement in understanding the genetic basis of fat deposition traits in sheep. In the selection of signal analysis methods, no differentiation is made between animals with long tails, short tails, or tailless characteristics. Similarly, no distinction is drawn between thin-tailed, short-thin-tailed. The classification is based on the amount of fat stored in the tail region, with individuals grouped accordingly into two categories: the fat-tailed group (encompassing sheep with fat rumps, fat tails, long fat tails, and short fat tails) and the thin-tailed group (including those with long thin tails, short thin tails, and generalized thin tails). These signals provide crucial insights into the evolutionary and artificial selection processes that have shaped the unique fatty-tail phenotype, which is prevalent in many sheep breeds, especially in arid and semi-arid regions. Fat stored in the tail is not randomly accumulated, but rather follows a precise biological logic. In extreme temperature fluctuations, it serves as both an insulator to maintain body temperature and a metabolic reservoir that can be broken down into energy and water through β-oxidation, a dual function critical for survival in harsh climates. This trait has been sculpted by millennia of natural selection, with genomic regions governing lipid synthesis and storage becoming enriched in populations native to arid zones. Concurrently, artificial selection has amplified these signals—ancient pastoralists prioritized individuals with larger tails, as they offered better meat quality and a reliable fat source for cooking and traditional medicine, creating genetic bottlenecks that further concentrated advantageous variants. In this study, we integrated 555 genomes and conducted a comprehensive analysis of selection signals between fat-tailed and thin-tailed sheep. 32 candidate genes were identified based on three methods: Fst-Pi, XP-CLR, and ROH. Genome-wide association analysis revealed that missense mutations (G/A and C/T) of *PDGFD* (Chr15: 3900312) and *BMP2* (Chr13: 48462350) were significantly associated with tail lipid deposition (Jin et al., 2025). Functional experiments have shown that the activation and expression of *PDGFD* mutants reduce fat deposition, while *BMP2* mutants promote the differentiation of preadipocytes and increase tail fat deposition. The two genes regulate tail fat development through complementary mechanisms: *PDGFD* encourages the expansion of adipose tissue, and *BMP2* regulates energy distribution. *GNAQ* is associated with lipid metabolism and reproductive traits, and its missense mutations (such as *GPR35* g.952,651 A>G) are significantly associated with tail fat weight in Hu sheep (Zhao et al., 2024). *SPAG17* is associated with extracellular matrix (ECM) remodeling and fibrosis, and may be involved in adipocyte homeostasis regulation in caudate tissue (Xu et al., 2023). *TBX15* is related to lipid metabolism and tail shape. It was listed as a candidate gene in the genomic difference analysis of Mongolian sheep (short fat tail) and Pamei sheep (long thin tail) (Li et al., 2024). In conclusion, the formation of sheep tail lipid is regulated by a multi-gene network. The functions of core genes (such as *PDGFD*, *BMP2*, *TBX15*) have been partially clarified, but the roles of other genes (such as *GLIS1*, *MSRB3*) still need to be further explored. These findings provide a theoretical basis for molecular breeding and the improvement of tail shape (Wang et al., 2025).

Furthermore, through Gene Ontology (GO) enrichment analysis of 32 differentially expressed genes, a total of 8 significantly enriched biological processes were identified (Figure 5; Table 1). These signaling pathways can be divided into three distinct functions: (1) Mesenchymal Cell Differentiation the Cellular Foundation of Fat Accumulation. The “mesenchymal cell differentiation” process, featuring key genes *BMP2*, *NOTCH1*, and *ALDH1A2*, emerges as a central driver of tail lipid deposition. Single-cell atlas analysis reveals that mesenchymal stem cells in the tail tissues of fat-tailed sheep exhibit a biased differentiation trajectory toward adipocytes, with *BMP2* acting as a critical switch to initiate adipogenic commitment. Notably, the laminin-mediated signaling pathway, highly expressed in Guangling large-tailed sheep and Hu sheep, reinforces this differentiation process by stabilizing the extracellular matrix (ECM) microenvironment, facilitating the transition of mesenchymal cells into mature adipocytes (Wang et al., 2025). This aligns with the observation that breeds with robust tail fat deposition show enhanced activity in mesenchymal cell lineage specification, highlighting the cellular origin of phenotypic differences; (2) Hormone Metabolic Networks systemic Regulators of Lipid Balance. Enriched GO terms related to “hormone metabolic processes” and “hormone level regulation” (encompassing *BMP2*, *MEGF8*, *NOTCH1*, and *PDGFD*) underscore the systemic regulation of tail fat deposition. These genes form a hormone-sensing network: *PDGFD* modulates insulin sensitivity in adipocytes, while *BMP2* interacts with thyroid hormone signaling to adjust lipid synthesis rates (Han et al., 2021). By fine-tuning hormone metabolism, this cluster indirectly coordinates energy allocation between tail fat storage and other physiological demands, ensuring adaptive responses to nutritional fluctuations—a mechanism particularly vital for sheep in resource-scarce arid regions; (3) Tissue Morphogenesis. Shaping the Structural Framework of Fat Depots. The enrichment of “tissue morphogenesis” terms reflects the structural remodeling required for large-scale fat accumulation. Single-cell analysis reveals that adipocytes in fat-tailed sheep secrete specific extracellular matrix (ECM) components, such as collagen subtypes, under the regulation of morphogenesis-related genes, shaping the architecture of tail adipose tissue. This structural adaptation allows for expanded lipid storage capacity, with distinct tissue morphologies (e.g., compact vs. diffuse fat distribution) corresponding to different tail shapes. The cell communication network, mediated by growth factor signals, coordinates this morphogenetic process, ensuring the synchronized expansion of fat depots and supporting the development of vasculature (Wang et al., 2025). Collectively, these findings underscore that a complex multi-gene network regulates sheep tail lipid deposition. While the functions of core genes such as *PDGFD*, *BMP2*, and *TBX15* have been partially elucidated, the roles of other candidates (e.g., *GLIS1* and *MSRB3*) remain to be characterized through functional assays, including gene knockout/overexpression studies and downstream pathway analyses. These results not only enhance our understanding of the genetic basis of fat-tailed phenotypes but also provide a theoretical framework for molecular breeding strategies that aim to tailor sheep traits to diverse agricultural and environmental needs (Wang et al., 2025).

## Conclusion

This study conducted a genome-wide analysis of 555 sheep, revealing the genetic structure of the sheep population and the genetic mechanism of tail fat deposition. Three methods identified thirty-two candidate genes related to caudal lipid deposition. For instance, *PDGFD* inhibits caudal lipid deposition, *BMP2* promotes the differentiation of preadipocytes, *GLIS1* affects the differentiation of mesodermal cells, and *TBX15* and *LIPE* are newly discovered genes. GO and KEGG analyses revealed that these genes were enriched in pathways such as mesenchymal cell differentiation and growth factor response, showing that fat deposition in sheep tails is regulated by a multi-gene network and providing a theoretical basis for molecular breeding and tail shape improvement of sheep.

## Data Availability

The data in this paper have been deposited in the Genome Variation Map (GVM) in National Genomics Data Center, Beijing Institute of Genomics, Chinese Academy of Sciences and China National Center for Bioinformation, under accession number GVM001174.
